# Preconditioning Contractions Suppress Muscle Pain Markers after Damaging Eccentric Contractions

**DOI:** 10.1155/2018/3080715

**Published:** 2018-10-14

**Authors:** Hiroshi Nagahisa, Kazumi Ikezaki, Ryotaro Yamada, Takashi Yamada, Hirofumi Miyata

**Affiliations:** ^1^Biological Sciences, Graduate School of Sciences and Technology for Innovation, Yamaguchi University, Yoshida 1677-1, Yamaguchi 753-8515, Japan; ^2^Graduate School of Health Sciences, Sapporo Medical University, Sapporo 060-8556, Japan

## Abstract

Inexperienced vigorous exercise, including eccentric contraction (ECC), causes muscle pain and damage. Similar prior light exercise suppresses the development of muscle pain (repeated-bout effect), but the molecular mechanisms behind this are not sufficiently understood. In this study, the influence of a nondamaging preconditioning ECC load (Precon) on muscle pain-related molecules and satellite cell-activating factors was investigated at the mRNA expression level. Nine-week-old male Wistar rats (*n*=36) were divided into 2 groups: a group receiving only a damaging ECC (100 contractions) load (non-Precon) and a group receiving a nondamaging ECC (10 contractions) load 2 days before receiving the damaging ECC load (Precon). ECC was loaded on the left leg, and the right leg was regarded as the intact control (CTL). The medial head of the gastrocnemius muscle from all rats was excised 2 or 4 days after the damaging ECC loading, and the relative mRNA expression levels of muscle pain- and satellite cell-related molecules were quantitated using real-time RT PCR. Precon suppressed increases in MHC-embryonic and MHC-neonatal mRNA expressions. Enhancement of HGF, Pax7, MyoD, and myogenin mRNA expression was also suppressed, suggesting that Precon decreased the degree of muscle damage and no muscle regeneration or satellite cell activation occurred. Similarly, increases in mRNA expression of muscle pain-related molecules (BKB_2_ receptor, COX-2, and mPGEC-1) were also suppressed. This study clearly demonstrated that at the mRNA level, prior light ECC suppressed muscle damage induced by later damaging ECC and promoted recovery from muscle pain.

## 1. Introduction

Inexperienced vigorous exercise, including eccentric contraction (ECC), induces muscle damage, edema, reduction of tension, limitation of the range of motion, and muscle pain. Regarding muscle pain, delayed onset muscle soreness (DOMS) persists for several days after exercise and generally resolves after several days [1–3]. ECC more effectively promotes muscular hypertrophy compared with concentric and isometric contractions [4, 5]. Therefore, ECC is considered to be efficient exercise for rehabilitation and training aiming at improvement of muscle function. On the other hand, ECC-induced symptoms, such as DOMS, may influence activity in daily life, rehabilitation, and motivation of athletes for daily training even though the symptoms are transient. However, it is also known that performing similar exercise several days before training reduces the grade of DOMS and damage and promotes recovery of reduced tension [2, 6, 7]. This effect of pre-exercise is referred to as the repeated-bout effect, and it has been reported to appear within 2 days to one week [6–8] and persist for 6 months [9]. However, the physiological mechanisms concerning the effects of pre-exercise on muscle pain and regeneration remain still unclear.

Experimentally, in a model of muscle pain of inflammation induced by formalin injection into the gastrocnemius muscle, mRNA expression of the B_2_ receptor of bradykinin (BK) represents several bioactivities, including induction of increases in muscle pain [10]. As this muscle pain is suppressed by administration of BKB_2_ receptor antagonist, the BKB_2_ receptor is considered to be involved in muscle pain [8, 10]. Other molecules involved in pain include prostaglandin (PG) E_2_. The protein and mRNA expression levels of an enzyme involved in PGE_2_ synthesis, cyclooxygenase-2 (COX-2), increase after ECC [11]. Muscle pain and inflammation after ECC and those induced by carrageenan are reduced by administration of a COX-2 inhibitor, and the level of the final product of COX-2, PGE_2_, simultaneously decreases [11, 12]. There are several isozymes of PGE synthase, which is the final enzyme of the PGE_2_ synthetic pathway, and microsomal PGE synthase-1 (mPGES-1) has been suggested to be closely associated with COX-2 [13]. Accordingly, it is likely that expression of COX-2 and mPGES-1 in the PGE_2_ synthetic pathway is involved in muscle pain via PGE_2_ production.

Although the BKB_2_ receptor or enzymes in the PGE_2_ synthetic pathway, such as COX-2 and mPGES-1, may be associated with muscle pain, the influence of pre-exercise on the expression of these muscle pain-related molecules is unclear. In this study, the influence of prior light nondamaging ECC (Precon) on muscle damage, muscle pain-related molecules, and muscle regeneration (satellite cell-activating factors) induced by later damaging ECC was examined at the mRNA expression level.

## 2. Materials and Methods

### 2.1. Animals and Experimental Protocol

All rat experiments were performed at Yamaguchi University and Sapporo Medical University and conducted in accordance with approved protocols by the Committee on Animal Experiments of Yamaguchi University (No. 290) and Sapporo Medical University (No. 16-077). Animal care was in accordance with institutional guidelines.

Male Wistar rats (9-week old, *n*=36) were supplied by Sanyo Labo Service (Sapporo, Japan). Rats were housed in an environmentally controlled room (24 ± 2°C, 12 h : 12 h light-dark cycle) and given food and water ad libitum. Rats were assigned to a Precon group and a non-Precon group (*n*=18 in each group). In all the groups, ECC was loaded on the left leg, and the right leg was regarded as intact control (CTL) of each experimental group. In the Precon group, the left plantar flexor muscles were exposed to Precon (i.e., 10 repeated ECC) 2 days prior to 100 repeated damaging ECC. Previous studies showed that muscle damage increase progressively with the number of forced ECC [14, 15]. In the preliminary experiments, we confirmed that the maximum isometric torque of the plantar flexor muscles was not reduced 48 hours after 10 repeated ECC (data not shown). The left muscles in the non-Precon group were exposed to damaging ECC without Precon. 0 (Immediately), 2, or 4 days after completion of damaging ECC, the planter flexor muscles were removed from each animal (*n*=6 in each group). The medial gastrocnemius muscles were used for further analyses.

### 2.2. Eccentric Contractions

Under isoflurane anesthesia, rats were placed supine on a platform and their left foot was secured in a foot plate connected to a torque sensor (S-14154, Takei Scientific Instruments, Tokyo, Japan) at an angle of 0° dorsiflexion (i.e., 90° relative to the tibia). ECC comprised forced dorsiflexion from 0° to 40° at 150°/s combined with neuromuscular electrical stimulation (ES). Plantar flexor muscles were stimulated supramaximally (45 V) via a pair of surface electrodes every 4 s. Stimulation parameters were set as follows: 1 ms monophasic rectangular pulse and 50 Hz stimulation frequency. The torque production was measured during ES.

### 2.3. Histochemical Analysis

Cryostat sections (10 *μ*m) were stained with hematoxylin and eosin (H&E). To obtain sections with Evans blue dye (EBD), rats were intraperitoneally injected with 1% (wt/vol) EBD solution (1 mg/10 g body wt) 24 hours before sacrifice. H&E and EBD images were obtained from the serial sections using a fluorescence microscope BIOREVO BZ-9000 (KEYENCE, Osaka, Japan).

### 2.4. Real-Time RT PCR

The procedure described in the previous study was used for real-time RT PCR analysis [16]. The muscle samples were homogenized with TRIzol reagent (Molecular Probes, Breda, Netherlands) to extract total RNA. Genomic DNA was removed from total RNA by treating for 30 min at 37°C with TURBO DNase (Ambion, Austin, USA). To synthesize first strand cDNA, DNase-treated RNA (0.5 *μ*g) was used with an Exscript RT reagent kit (Takara, Tokyo, Japan). Subsequently, the SYBR Green PCR Master Mix protocol in the StepOne Plus Real-Time PCR system (Applied Biosystems Japan, Tokyo, Japan) was used for real-time PCR analysis of cDNA products.

The amplification program was composed of an initial denaturation step at 95° for 10 min, 40 cycles of denaturation at 95° for 30 sec, and annealing/extension at 58° for 1 min. Glyceraldehyde-3-phosphate dehydrogenase (GAPDH) was used to estimate an internal control. The normalization of each mRNA was calculated from GAPDH by subtracting the cycle threshold (*C*_t_) value of GAPDH from the *C*_t_ value of the target gene (Δ*C*_t_ (target)). The relative expression of the target gene was calculated as the relative quantification value for the CTL value. No nonspecific amplification in cDNA samples was detected by dissociation curve analysis after the relative expression.

Table 1 shows the sequences of the specific primers used in the present study. Primer Express software (Applied Biosystems Japan) was used for design of each PCR primer. The oligonucleotides were purchased from FASMAC (FASMAC, Kanagawa, Japan).

### 2.5. Statistical Analyses

All values are expressed as mean ± standard error (SE). Differences in mRNA expressions between groups were compared using one-way ANOVA followed by *t*-test with Bonferroni adjustment. Statistical significance was set at *P* < 0.05.

## 3. Results

### 3.1. Histochemical Properties of Skeletal Muscle after Damaging ECC

**Figure 1:**
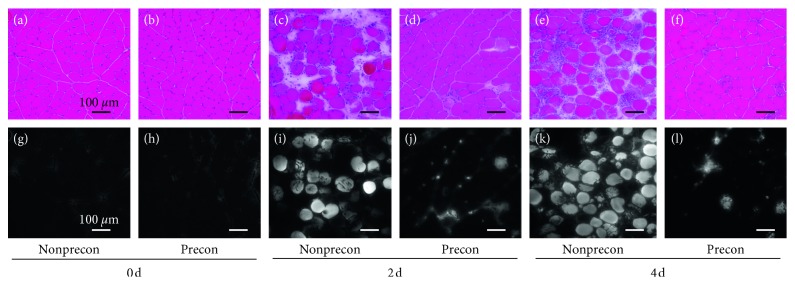
Photomicrograph of hematoxylin and eosin (a–f) and Evans blue dye (g–l) staining on sections of the left medial gastrocnemius muscles after damaging eccentric contractions.

There was not obvious histopathological alteration in sections of H&E at 0 day between non-Precon and Precon groups (Figures 1 and 1). In sections of H&E at 2 and 4 days after damaging ECC, a number of swollen myofibers with rounded shape and many inflammatory cells surrounding them were observed in the non-Precon group (Figures 1 and 1), while these unfavorable changes dramatically were suppressed in the Precon group (Figures 1 and 1).

In agreement with these results, EBD-positive fibers which indicate development of membrane damage were not observed in 0 day (Figures 1 and 1), increased at 2 and 4 days after damaging ECC in the non-Precon group (Figures 1 and 1), and little increased during the same period in the Precon group (Figures 1 and 1).

### 3.2. Expression of Skeletal Muscle Damage- and Regeneration-Related Factors mRNA

**Figure 2:**
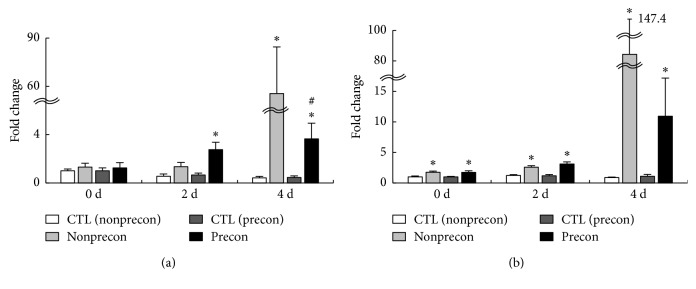
Time course changes in relative expression of MHC-embryonic (a) and MHC-neonatal (b) mRNA. The mRNA expression of each time point was calculated as x-fold change from each CTL value at 0 d. CTL indicates intact right muscle of each experimental group. Values are means ± SE. ^*∗*^significant differences (*P* < 0.05) as compared with each CTL value. ^#^significant differences (*P* < 0.05) as compared with each non-Precon value.

The expression level was presented as a value relative to that in CTL muscles of each group at 0 day. MHC-embryonic and MHC-neonatal are expressed during the regeneration process after muscle damage. At 2 days after damaging ECC, the expression level of MHC-embryonic mRNA was significantly increased in the Precon group (Figure 2(a)). At 4 days after damaging ECC, both the non-Precon and Precon groups showed significant increase in MHC-embryonic mRNA expression, whereas the expression of MHC-embryonic in the non-Precon group was significantly greater than in the Precon group.

The expression level of MHC-neonatal, at 0, 2, and 4 days after damaging ECC, was significantly increased in both the non-Precon and Precon groups (Figure 2(b)). However, there was no difference between the non-Precon and Precon groups.

**Figure 3:**
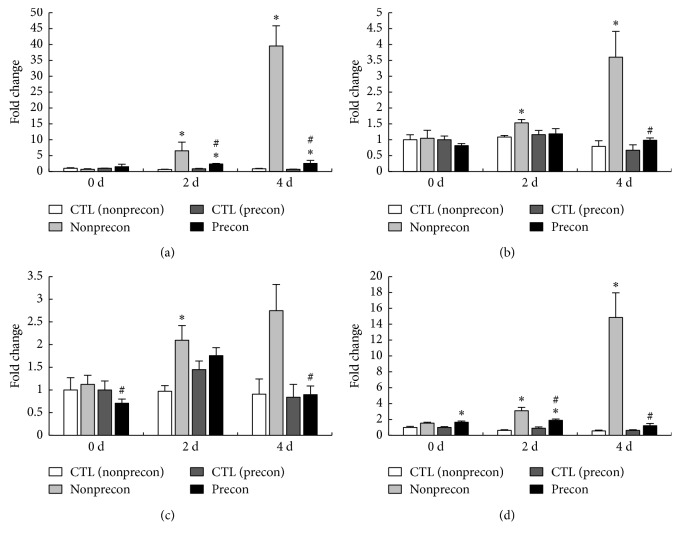
Time course changes in relative expression of HGF (a), Pax7 (b), MyoD (c), and myogenin (d) mRNA. The mRNA expression of each time point was calculated as x-fold change from each CTL value at 0 d. CTL indicates intact right muscle of each experimental group. Values are means ± SE. ^*∗*^significant differences (*P* < 0.05) as compared with each CTL value. ^#^significant differences (*P* < 0.05) as compared with each non-Precon value.

The expression level of HGF mRNA, which is a well-studied activation factor for satellite cells, was significantly increased in the Precon group at 2 and 4 days after damaging ECC (Figure 3(a)). In the non-Precon group, the mRNA expression of HGF was significantly increased at 2 and 4 days after damaging ECC. However, at 2 and 4 days after damaging ECC, the expression level of HGF mRNA was significantly lower in the Precon group compared with the non-Precon group.

The mRNA expression level of Pax7, which is expressed in activation and proliferation state of satellite cells, was significantly increased only in the nonPrecon group at 2 and 4 days after damaging ECC (Figure 3(b)). In addition, at 4 days after damaging ECC, the expression of Pax7 mRNA was significantly lower in the Precon group compared with non-Precon group.

The mRNA expression level of MyoD regulating proliferation and differentiation of satellite cells was significantly increased in the non-Precon group 2 days after damaging ECC (Figure 3(c)). At 4 days after damaging ECC, the expression level of MyoD mRNA in the Precon group was significantly lower than in the non-Precon group.

The mRNA expression level of myogenin, which is expressed in the differentiation state of satellite cells, was significantly increased in the Precon group at 0 and 2 days after damaging ECC (Figure 3(d)). In the non-Precon group, at 2 and 4 days after damaging ECC, the mRNA expression level of myogenin was significantly increased. Furthermore, the expression level of myogenin mRNA was significantly lower in the Precon group compared with non-Precon group at 2 and 4 days after damaging ECC.

### 3.3. The Expression of Muscle Pain-Related Molecules mRNA

**Figure 4:**
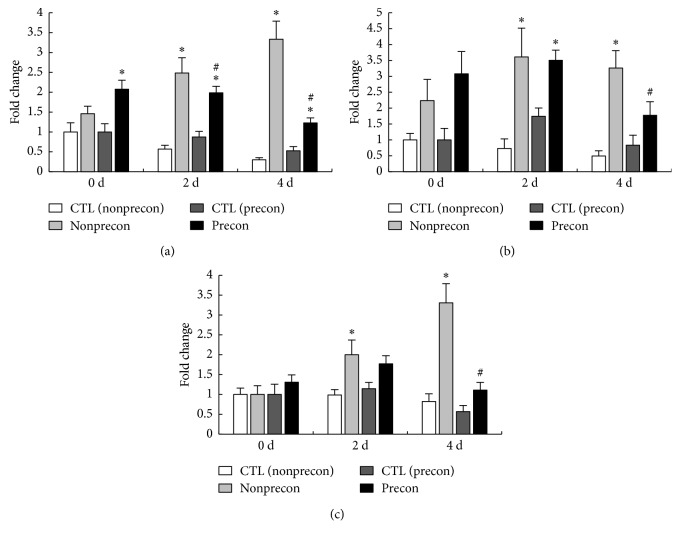
Time course changes in relative expression of BKB_2_ receptor (a), COX-2 (b), and mPGES-1 (c) mRNA. The mRNA expression of each time point was calculated as x-fold change from each CTL value at 0 d. CTL indicates intact right muscle of each experimental group. Values are means ± SE. ^*∗*^significant differences (*P* < 0.05) as compared with each CTL value. ^#^significant differences (*P* < 0.05) as compared with each non-Precon value.

The expression level of BKB_2_ receptor mRNA in the Precon group was significantly increased at 0, 2, and 4 days after damaging ECC (Figure 4(a)). In the non-Precon group, the mRNA expression of BKB_2_ receptor was significantly increased at 2 and 4 days after damaging ECC. However, at 2 and 4 days after damaging ECC, the expression level of BKB_2_ receptor mRNA was significantly lower in the Precon group compared with the non-Precon group.

The mRNA expression level of COX-2 in the Precon and non-Precon group was significantly increased at 2 days after damaging ECC (Figure 4(b)). However, at 4 days after damaging ECC, the expression of COX-2 was significantly increased only in the non-Precon group and its expression in the Precon group was significantly lower than in the non-Precon group.

The expression level of mPGES-1 in the non-Precon group was significantly increased at 2 and 4 days after damaging ECC, whereas there was no marked alteration in the Precon group (Figure 4(c)). In addition, at 4 days after damaging ECC, the expression level of mPGES-1 mRNA was significantly lower in the Precon group compared with the non-Precon group.

## 4. Discussion

The influence of prior light nondamaging ECC on muscle damage, muscle pain, and muscle regeneration-related molecules induced by later damaging ECC was investigated. In fact, Precon suppressed histopathological alteration induced by damaging ECC, corresponding to our previous results [17]. Furthermore, Precon suppressed increases in MHC-embryonic and MHC-neonatal mRNA expressions, suggesting a decrease in the degree of muscle damage. Similarly, increases in mRNA expression of HGF, Pax7, MyoD, and myogenin were suppressed by Precon, suggesting that Precon reduced the degree of muscle damage, negating the need for muscle regeneration. Enhancement of mRNA expression of muscle pain-related molecules was also suppressed. This study demonstrated that prior light ECC reduces muscle damage induced by later damaging ECC and promotes recovery from muscle pain at the mRNA level.

In this study, we did not confirm the effect of Precon itself on mRNA expression. However, most of the mRNA expressions including muscle pain-related molecules in the non-Precon group did not significantly increase compared with its control and Precon groups at 0 day (2 days after Precon). Considering that the Precon itself (10 repeated ECC) has less impact than non-Precon (100 repeated ECC), the effect of Precon itself on mRNA expression was speculated to be little at least 0 and 2 days after the Precon.

### 4.1. Muscle Damage and Regeneration Induced by Eccentric Contraction

Skeletal muscle expresses developmental MHC, such as MHC-embryonic and MHC-neonatal, in the recovery course after muscle damage, in addition to mature MHC, such as MHC I, IIa, IIx, and IIb [18, 19]. These developmental MHCs are replaced by mature MHC during recovery after muscle damage. MHC-embryonic and MHC-neonatal mRNA expression increased after damaging ECC, but the degree of the increase in MHC-embryonic at 4 days after damaging ECC was reduced by Precon. Muscle satellite cells play an important role in muscle regeneration after muscle damage. Satellite cells are localized between the basement and plasma membranes, but they are normally in the resting state and express Pax7 [20]. When satellite cells are stimulated by muscle damage, they are activated, and they proliferate, differentiate, and supply new muscle fiber and nuclei [20]. Some cells then return to the resting state [20]. Many studies have demonstrated that growth factors such as HGF are associated with activation and proliferation of myoblasts [21]. Furthermore, MyoD and myogenin are expressed when satellite cells are activated, and they proliferate, or differentiate [20]. In the rats that received only damaging ECC, HGF, Pax7, MyoD, and myogenin mRNA expression increased after damaging ECC, suggesting a functional muscle regeneration mechanism, whereas these increases were not observed in the Precon group, suggesting that prior Precon reduced the degree of muscle damage, leading to only a slight need for muscle regeneration. Damage by vigorous ECC has been clarified by indirect analysis (serum creatine kinase) [1, 2] and histological analysis [22–24]. In our study, the muscle was damaged in the group only with damaging ECC, confirming that Precon markedly reduced the degree of damage at the mRNA expression level.

### 4.2. Muscle Pain Markers Induced by Eccentric Contraction

PGE_2_ increases in skeletal muscle during dynamic exercise, but it does not increase during static exercise [25]. An enzyme in the PGE_2_ synthetic pathway, COX-2, is highly involved in muscle pain. In fact, COX-2 inhibitors can reduce muscle pain when it is applied before lengthening contraction [11, 12]. In addition, the final enzyme of PGE_2_ synthesis, mPGES-1, also produces PGE_2_ from COX-2-derived metabolites [13] and is also involved in PGE_2_-induced pain and inflammation [26]. Therefore, COX-2 and mPGES-1 may induce muscle pain through PGE_2_ production. In inflammation, COX-2 is expressed in neutrophils, macrophages, endothelial cells, and muscle nuclei/muscle satellite cells [11, 27, 28]. mPGES-1 has been reported to be localized in macrophages and fibroblasts during inflammation [29, 30]. In the present study, Precon suppressed COX-2 and mPGES-1 mRNA expression, indicating decreased PGE2 production. Accordingly, muscle pain due to PGE_2_ may have been reduced in the Precon group compared with in the non-Precon group.

Eccentric exercise elevates the blood [31] and interstitial BK levels in skeletal muscle and tendon tissue [32]. There are 2 types of BK receptors: BKB_1_ and BKB_2_. The BKB_1_ receptor is expressed when tissue is damaged and contributes to chronic inflammation, whereas the BKB_2_ receptor is constantly expressed and considered to be involved in acute inflammation [33–35]. BKB_2_ receptor mRNA expression was decreased by Precon 2 and 4 days after damaging ECC. In a previous study, muscle pain developed in the intact leg from 1 to 4 days after eccentric contraction, but when a BKB_2_ receptor antagonist was administered before eccentric contraction, later muscle pain was suppressed, whereas the BKB_1_ receptor antagonist exerted no muscle pain-inhibitory effects [36]. In an inflammation model induced by intramuscular injection of formalin, both BKB_1_ and B_2_ receptor antagonists demonstrated muscle pain-inhibitory effects from immediately after formalin injection to 7 hours later, but only the B_2_ receptor antagonist reduced IL-6 mRNA expression [10]. IL-6 is an inflammatory marker that has been suggested to contribute to muscle pain [37, 38]. Thus, the BKB_2_ receptor may be more closely involved in muscle pain from inflammation compared with the B_1_ receptor. In addition, administration of a BKB_2_ receptor antagonist 30 minutes after ECC did not suppress muscle pain, suggesting that the BKB_2_ receptor functions in the development of muscle pain rather than the maintenance of muscle pain [36]. In this study, BKB2 receptor mRNA expression showed significant increase at 0 day (immediately after damaging ECC). However, BKB2 receptor mRNA expression of the non-Precon group gradually increased while those of the Precon group showed gradual decrease, suggesting that the peak of pain marker shifted to the left in the Precon group. Although we could not prove whether the peak shift of BKB2 receptor mRNA expression directly relate to muscle pain, the mRNA expression of the Precon group was suppressed in accordance with other muscle pain-related molecules and histopathological damage at 2 and 4 days after damaging ECC.

In summary, we found that the BKB2 receptor which plays an important role in muscle pain significantly increased after eccentric contraction and the expression of mRNA was suppressed by prior low-load ECC in relatively early stage.

### 4.3. Influence of Preconditioning on Training Effect

ECC- and pre-exercise-induced muscle damage, muscle regeneration, and muscle pain were investigated. Our results suggest that prior light ECC reduces muscle damage caused by later damaging ECC and accompanying muscle regeneration and suppresses muscle pain at the mRNA expression level. Based on these findings, in order to reduce exercise-induced muscle pain, prior light exercise may be preferable. However, to improve muscle function, prior light exercise may reduce the later training effects because Precon suppressed mRNA expression of a muscle satellite cell marker, Pax7, which plays an important role in muscular hypertrophy, as well as MyoD and myogenin, which are involved in proliferation and differentiation of satellite cells [20]. In addition, the importance of COX-2 for muscular hypertrophy has been reported using a compensatory muscular hypertrophy model with a COX-2 inhibitor [28]. Thus, suppression of COX-2 expression and activity may be a good method to reduce muscle pain, but COX-2 expression and activity may be necessary when the objective is muscular hypertrophy. Further studies on the long-term effects are necessary.

## Figures and Tables

**Table 1:** Real-time RT PCR primer sequences.

Gene	Accession number		Sequence	Primer position
GAPDH	NM_017008.4	Forward	GCTCTCTGCTCCTCCCTGTTC	4–24
		Reverse	GAGGCTGGCACTGCACAA	44–61
MHC-embryonic	NM_012604.1	Forward	CTTCAAACTGAAAAACGCCTATGA	4536–4559
		Reverse	GTTCTAAGTTCTTATTCTCTCGCTTCACA	4584–4612
MHC-neonatal	NM_001100485.1	Forward	ATCAGTGCCAATCCCTTGCT	742–761
		Reverse	CCAAAGCGAGGAGTTGTCA	795–815
HGF	NM_017017.2	Forward	AAAACTACATGGGCAACTTATCCAA	1335–1359
		Reverse	ATGACGGTGTAAATCCTCCATATTC	1396–1420
Pax7	NM_001191984.1	Forward	AAAAGATTGAGGAGTATAAGAGGGAGAA	347–374
		Reverse	GCCGGTCCCGGATTTC	394–409
MyoD	NM_176079.1	Forward	GACGGCTCTCTCTGCTCCTTT	259–279
		Reverse	AGTCGAAACACGGATCATCATAGA	296–319
Myogenin	M24393.1	Forward	GACCCTACAGGTGCCCACAA	604–623
		Reverse	CCGTGATGCTGTCCACGAT	643–661
BKB_2_ receptor	M59967.2	Forward	GAGCTTGAAGCATCCTAGGGAAT	1543–1565
		Reverse	CGCTTATGCCGTGAGACAAGA	1583–1603
COX-2	U03389.1	Forward	GGCAAAGGCCTCCATTGAC	1423–1441
		Reverse	GCGTTTGCGGTACTCATTGA	1470–1489
mPGES-1	NM_021583.3	Forward	TGCTCCCCGCCTTTCTG	78–94
		Reverse	CCACCGCGTACATCTTGATG	115–134

GAPDH, glyceraldehydes-3-phosphate dehydrogenase; MHC, myosin heavy chain; HGF, hepatocyte growth factor; Pax7, paired box transcription factor-7; MyoD, myogenic determination factor; BKB_2_, bradykinin B_2_ receptor; COX-2, cyclooxygenase 2; mPGES-1, microsomal prostaglandin E synthase-1.

## Data Availability

The data used to support the findings of this study are available from the corresponding author upon request.

## References

[1] Nelson N. (2013). Delayed onset muscle soreness: is massage effective?. *Journal of Bodywork and Movement Therapies*.

[2] Chen T. C., Chen H. L., Pearce A. J., Nosaka K. (2012). Attenuation of eccentric exercise-induced muscle damage by preconditioning exercises. *Medicine & Science in Sports & Exercise*.

[3] Hyldahl R. D., Hubal M. J. (2014). Lengthening our perspective: morphological, cellular, and molecular responses to eccentric exercise. *Muscle & Nerve*.

[4] Roig M., O’Brien K., Kirk G. (2009). The effects of eccentric versus concentric resistance training on muscle strength and mass in healthy adults: a systematic review with meta-analysis. *British Journal of Sports Medicine*.

[5] Hedayatpour N., Falla D. (2015). Physiological and neural adaptations to eccentric exercise: mechanisms and considerations for training. *BioMed Research International*.

[6] Lavender A. P., Nosaka K. (2008). A light load eccentric exercise confers protection against a subsequent bout of more demanding eccentric exercise. *Journal of Science and Medicine in Sport*.

[7] Maeo S., Yamamoto M., Kanehisa H., Nosaka K. (2017). Prevention of downhill walking-induced muscle damage by non-damaging downhill walking. *PLoS One*.

[8] Urai H., Murase S., Mizumura K. (2013). Decreased nerve growth factor upregulation is a mechanism for reduced mechanical hyperalgesia after the second bout of exercise in rats. *Scandinavian Journal of Medicine & Science in Sports*.

[9] Nosaka K., Sakamoto K., Newton M., Sacco P. (2001). How long does the protective effect on eccentric exercise-induced muscle damage last?. *Medicine & Science in Sports & Exercise*.

[10] Meotti F. C., Campos R., da Silva K., Paszcuk A. F., Costa R., Calixto J. B. (2012). Inflammatory muscle pain is dependent on the activation of kinin B_1_ and B_2_ receptors and intracellular kinase pathways. *British Journal of Pharmacology*.

[11] Murase S., Terazawa E., Hirate K. (2013). Upregulated glial cell line-derived neurotrophic factor through cyclooxygenase-2 activation in the muscle is required for mechanical hyperalgesia after exercise in rats. *Journal of Physiology*.

[12] Zhang Y., Shaffer A., Portanova J., Seibert K., Isakson P. C. (1997). Inhibition of cyclooxygenase-2 rapidly reverses inflammatory hyperalgesia and prostaglandin E2 production. *Journal of Pharmacology and Experimental Therapeutics*.

[13] Samuelsson B., Morgenstern R., Jakobsson P. J. (2007). Membrane prostaglandin E synthase-1: a novel therapeutic target. *Pharmacological Reviews*.

[14] Hesselink M. K., Kuipers H., Geurten P., Van Straaten H. (1996). Structural muscle damage and muscle strength after incremental number of isometric and forced lengthening contractions. *Journal of Muscle Research & Cell Motility*.

[15] Willems M. E., Stauber W. T. (2009). The effect of number of lengthening contractions on rat isometric force production at different frequencies of nerve stimulation. *Acta Physiologica*.

[16] Nagahisa H., Okabe K., Iuchi Y., Fujii J., Miyata H. (2016). Characteristics of skeletal muscle fibers of SOD1 knockout mice. *Oxidative Medicine and Cellular Longevity*.

[17] Yamada R., Himori K., Tatebayashi D. (2018). Preconditioning contractions prevent the delayed onset of myofibrillar dysfunction after damaging eccentric contractions. *Journal of Physiology*.

[18] Schiaffino S., Rossi A. C., Smerdu V., Leinwand L. A., Reggiani C. (2015). Developmental myosins: expression patterns and functional significance. *Skeletal Muscle*.

[19] Ciciliot S., Schiaffino S. (2010). Regeneration of mammalian skeletal muscle. Basic mechanisms and clinical implications. *Current Pharmaceutical Design*.

[20] Dumont N. A., Wang Y. X., Rudnicki M. A. (2015). Intrinsic and extrinsic mechanisms regulating satellite cell function. *Development*.

[21] Yamada M., Tatsumi R., Yamanouchi K. (2010). High concentrations of HGF inhibit skeletal muscle satellite cell proliferation in vitro by inducing expression of myostatin: a possible mechanism for reestablishing satellite cell quiescence in vivo. *American Journal of Physiology-Cell Physiology*.

[22] Kano Y., Masuda K., Furukawa H., Sudo M., Mito K., Sakamoto K. (2008). Histological skeletal muscle damage and surface EMG relationships following eccentric contractions. *Journal of Physiological Sciences*.

[23] Sudo M., Kano Y. (2009). Myofiber apoptosis occurs in the inflammation and regeneration phase following eccentric contractions in rats. *Journal of Physiological Sciences*.

[24] Hyldahl R. D., Olson T., Welling T., Groscost L., Parcell A. C. (2014). Satellite cell activity is differentially affected by contraction mode in human muscle following a work-matched bout of exercise. *Frontiers in Physiology*.

[25] Karamouzis M., Langberg H., Skovgaard D., Bulow J., Kjaer M., Saltin B. (2001). In situ microdialysis of intramuscular prostaglandin and thromboxane in contracting skeletal muscle in humans. *Acta Physiologica Scandinavica*.

[26] Dallaporta M., Pecchi E., Thirion S., Jean A., Troadec J. D. (2010). Toward the management of inflammation: recent developments of mPGES-1 inhibitors. *Recent Patents on CNS Drug Discovery*.

[27] Bachawaty T., Washington S. L., Walsh S. W. (2010). Neutrophil expression of cyclooxygenase 2 in preeclampsia. *Reproductive Sciences*.

[28] Novak M. L., Billich W., Smith S. M. (2009). COX-2 inhibitor reduces skeletal muscle hypertrophy in mice. *American Journal of Physiology-Regulatory, Integrative and Comparative Physiology*.

[29] Korotkova M., Helmers S. B., Loell I. (2008). Effects of immunosuppressive treatment on microsomal prostaglandin E synthase 1 and cyclooxygenases expression in muscle tissue of patients with polymyositis or dermatomyositis. *Annals of the Rheumatic Diseases*.

[30] Korotkova M., Jakobsson P. J. (2011). Microsomal prostaglandin e synthase-1 in rheumatic diseases. *Frontiers in Pharmacology*.

[31] Blais C., Adam A., Massicotte D., Péronnet F. (1999). Increase in blood bradykinin concentration after eccentric weight-training exercise in men. *Journal of Applied Physiology*.

[32] Langberg H., Bjørn C., Boushel R., Hellsten Y., Kjaer M. (2002). Exercise-induced increase in interstitial bradykinin and adenosine concentrations in skeletal muscle and peritendinous tissue in humans. *Journal of Physiology*.

[33] Hamza M., Wang X. M., Adam A. (2010). Kinin B1 receptors contributes to acute pain following minor surgery in humans. *Molecular Pain*.

[34] Su J. B. (2014). Different cross-talk sites between the renin-angiotensin and the kallikrein-kinin systems. *Journal of the Renin-Angiotensin-Aldosterone System*.

[35] Couture R., Harrisson M., Vianna R. M., Cloutier F. (2001). Kinin receptors in pain and inflammation. *European Journal of Pharmacology*.

[36] Murase S., Terazawa E., Queme F. (2010). Bradykinin and nerve growth factor play pivotal roles in muscular mechanical hyperalgesia after exercise (delayed-onset muscle soreness). *Journal of Neuroscience*.

[37] Dina O. A., Green P. G., Levine J. D. (2008). Role of interleukin-6 in chronic muscle hyperalgesic priming. *Neuroscience*.

[38] Manjavachi M. N., Motta E. M., Marotta D. M., Leite D. F., Calixto J. B. (2010). Mechanisms involved in IL-6-induced muscular mechanical hyperalgesia in mice. *Pain*.

